# Strong Generalized Speech Emotion Recognition Based on Effective Data Augmentation

**DOI:** 10.3390/e25010068

**Published:** 2022-12-30

**Authors:** Huawei Tao, Shuai Shan, Ziyi Hu, Chunhua Zhu, Hongyi Ge

**Affiliations:** 1 Key Laboratory of Food Information Processing and Control, Ministry of Education, Henan University of Technology, Zhengzhou 450001, China; 2 Henan Engineering Laboratory of Grain IOT Technology, Henan University of Technology, Zhengzhou 450001, China

**Keywords:** speech emotion recognition, data augmentation, multi-channel feature extractor, Wasserstein distance, feature distributions, speaker-invariant emotional representations

## Abstract

The absence of labeled samples limits the development of speech emotion recognition (SER). Data augmentation is an effective way to address sample sparsity. However, there is a lack of research on data augmentation algorithms in the field of SER. In this paper, the effectiveness of classical acoustic data augmentation methods in SER is analyzed, based on which a strong generalized speech emotion recognition model based on effective data augmentation is proposed. The model uses a multi-channel feature extractor consisting of multiple sub-networks to extract emotional representations. Different kinds of augmented data that can effectively improve SER performance are fed into the sub-networks, and the emotional representations are obtained by the weighted fusion of the output feature maps of each sub-network. And in order to make the model robust to unseen speakers, we employ adversarial training to generalize emotion representations. A discriminator is used to estimate the Wasserstein distance between the feature distributions of different speakers and to force the feature extractor to learn the speaker-invariant emotional representations by adversarial training. The simulation experimental results on the IEMOCAP corpus show that the performance of the proposed method is 2–9% ahead of the related SER algorithm, which proves the effectiveness of the proposed method.

## 1. Introduction

Speech emotion recognition (SER) plays an important role in Human-Computer Interaction (HCI) systems, and it has become increasingly involved in a wide variety of industrial applications. SER, for instance, can be used to detect the presence and severity of a patient’s distress without requiring any intervention from a human [1]. An intelligent customer service system in the call center will transfer a call to a human customer service representative if it recognizes that the customer expresses a negative emotion [2]. In the field of education, the use of SER can greatly improve teaching and learning outcomes [3]. It is of great practical importance to conduct research for SER to make HCI more intelligent and humane.

Deep learning has become a viable technical solution for SER, and SER methods based on deep learning have achieved better performance in a variety of scenarios. The training of high-performing models requires a large number of samples. Manually labeling emotion labels is, however, time-consuming and costly, limiting the size of the existing emotion corpus. SER is limited by the lack of a large-scale labeled emotion corpus. Researchers have attempted to solve the problem of small emotional corpus samples using data augmentation methods in recent years, with some success. Aldeneh et al. [4] varied the speed of the speech, creating two additional speech copies of 0.9× and 1.1× speed to increase the size of the training data and achieved a 2.8% recognition rate improvement on the MSP-IMPROV [5] corpus. Li et al. [6] also used variable speed speech to expand the corpus and achieved better performance in the speaker-independent case. Braunschweiler et al. [7] used speed augmentation and volume perturbation, and the experimental results showed that data augmentation is still effective in improving performance in the case of cross-corpus recognition. The authors of [8] also reported significant performance gains using data augmentation which is noise injection and volume perturbation in the EMODB [9] corpus. Liu et al. [10] used SpecAugment, a data augmentation algorithm proposed by Google [11], to enrich a few classes of emotional speech samples to balance the corpus. To some extent, these works fill the research gap regarding data augmentation in the SER field. However, fewer data augmentation methods have been explored, and no scholars have yet made detailed and specific studies on acoustic-based data enhancement methods in the field of SER.

Besides directly altering the acoustic properties of the original speech to create new speech to augment the training data, some scholars have also used GANs [12] to address the problem of data scarcity in SER. In [13], the authors used Balancing GAN [14] to generate speech spectrograms of target categories to increase the number of training samples. Since it is difficult for generators to generate high-dimensional samples directly, Yi et al. [15] proposed ADAN (Adversarial Data Augmentation Network), which combines Autoencoder techniques with GAN to generate low-dimensional emotional vectors in latent space. However, it is difficult to train a generator that is able to generate accurate emotional samples from the target categories due to the confusion among some specific emotions [10] and the possibility of mode collapse of the generators [16].

Log–Mel spectrograms have the advantage of high correlation and are widely used in various speech tasks [17,18,19,20]. We also use log–Mel spectrograms as the input to the proposed end-to-end SER model. In this paper, we first investigate the impact of acoustic-based data augmentation methods on SER through a simple model, and, based on that, we propose a strong generalized speech emotion recognition model based on effective data augmentation. There are three components to the model: a feature extractor, an emotion classifier, and a discriminator. We utilize a multi-channel feature extractor consisting of multiple sub-networks to extract emotional representations under multiple data augmentations. The discriminators were used to estimate the Wasserstein distance [21] between different speakers’ emotional representations. By reducing this distance through adversarial training, the feature extractor can learn the speaker-invariant emotional representations. The main contributions of this paper are three points:

- The effectiveness of acoustic-based data augmentation methods is evaluated in SER.
- There is a feature extractor architecture proposed that can make better use of data augmentation methods, which consists of multiple sub-networks, and a model weight parameter sharing strategy is applied among the sub-networks. The output feature maps of each sub-network are fused to generate emotional representations.
- In order to generalize the emotional representations, the Wasserstein distance is used to measure the distribution of emotional representations among speakers. The distribution of representations is approximated in hidden space by adversarial training. In this way, the feature extractor learns the speaker-invariant emotional representations.

## 2. Proposed Method

### 2.1. Strong Generalized Speech Emotion Recognition Model Based on Effective Data Augmentation

**Figure 1:**
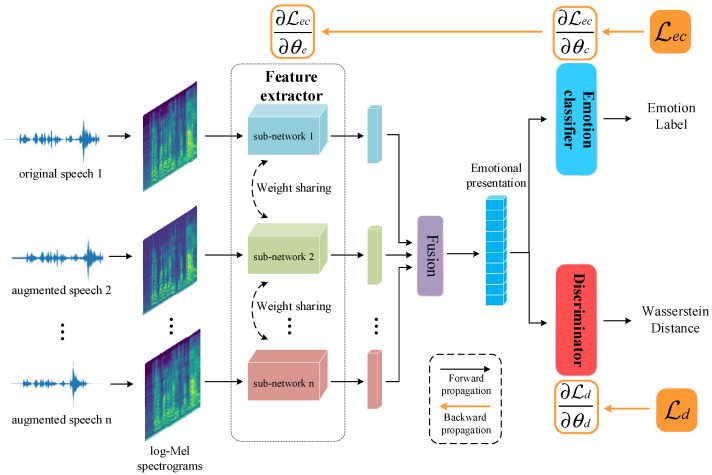
The structure of the proposed strong generalized speech emotion recognition model based on effective data augmentation.

The structure of the proposed model is shown in Figure 1. There are three modules in the model: a feature extractor, an emotion classifier, and a discriminator. Log–Mel spectrograms of the original and augmented speech are fed into a multi-channel feature extractor, which is responsible for extracting the emotional representations in the spectrograms. Emotional representations are input to the emotion classifier for classification and to the discriminator for estimating the Wasserstein distance. In the emotion classifier, there are two fully connected layers (256:64, 64:4) as well as a softmax layer and dropout set to 0.5. In the discriminator, there are two fully connected layers (265:64, 64:16, 16:1). The activation function used in these two modules is the Rectified Linear Unit (ReLU). A detailed description of the feature extractor is provided in the following Section 2.3. After the model has been trained, the feature extractor and emotion classifier can form a complete SER system.

### 2.2. Data Augmentation

**Figure 2:**
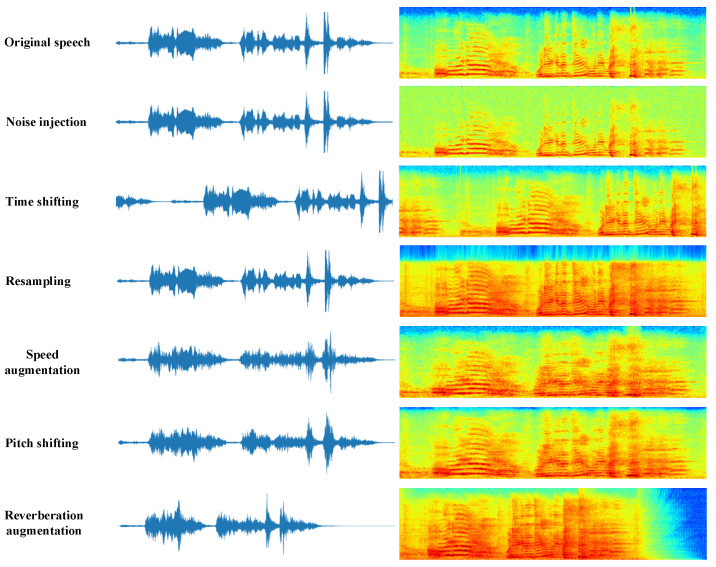
Speech waveforms and spectrograms with different data augmentation methods.

Following a summary of acoustic data augmentation methods previously applied to the SER and other speech tasks, six acoustic data augmentation methods were selected for analysis: speed augmentation [4,6,7], noise injection [8], time shifting [22], resampling [23], pitch shifting [24], and reverberation augmentation [25]. Figure 2 shows speech waveforms and spectrograms with different data augmentation methods. It should be noted that speed augmentation and reverberation augmentation change the length of the speech, but we unify the length of their waveforms and spectrograms in Figure 2 for the sake of comparison. A detailed description and implementation of each type of data augmentation are provided in the following Section 3.2.

### 2.3. Feature Extractor

**Figure 3:**
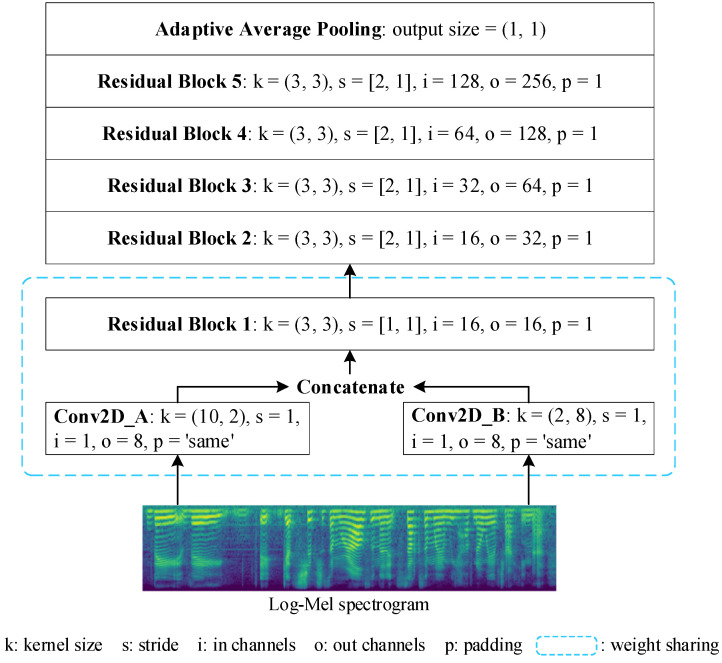
The sub-network structure in the feature extractor used by the proposed model.

The feature extractor consists of n sub-networks with the same parameter settings, and each sub-network receives log–Mel spectrograms for different augmented speech as inputs. The Residual Network [26], which is commonly used in SER, is selected as the main part of the sub-network in this study based on a literature review [27,28,29,30]. Figure 3 shows a specific sub-network setup where log–Mel spectrograms are fed into two parallel convolution layers with convolutional kernels of (10, 2) and (2, 8), respectively. Such a convolution kernel setup can fully extract the time and frequency domain information of log–Mel spectrograms [31]. Five consecutive residual blocks [26] are used to extract deep emotional information from the concatenated outputs of the two convolutional layers described above. The feature map size is eventually compressed using adaptive average pooling to retain only relevant information. In order to facilitate sharing of some learned knowledge, such as low-level acoustic features, between channels of the feature extractor while accelerating the convergence speed, a model weight parameter sharing strategy is applied in Conv2D_A, Conv2D_B and Residual_Block_1 between each sub-network. The output feature maps of each sub-network are fused into the emotional representations needed for the subsequent classification and metric tasks based on their weighting coefficient. The emotional representation EmoRep is calculated by:

(1) $$EmoRep=\alpha_{1}*f_{\theta 1}(x_{1})+\alpha_{2}*f_{\theta 2}(x_{2})+\ldots \ldots +\alpha_{n}*f_{\theta n}(x_{n})$$

where $\alpha_{k}$ is the weighting coefficient, and $f_{\theta k}$ denotes the function of a sub-network.

### 2.4. Measuring Distance of Emotional Representation Distribution

The Wasserstein distance was used to measure the distance between the distributions of emotional representations among the speakers. In high-dimensional space, if two distributions do not overlap or the overlap can be ignored, the KL and JS divergence do not reflect the distance between distributions or provide the gradient. In [21], the authors solved this problem by using the Wasserstein distance rather than the KL and JS divergences in the original GAN. Due to the superiority of the Wasserstein distance as a distribution measure, it is used in this paper to measure the distance between emotional representations. Given probability distributions ${\mathbb{P}}_{1}$ and ${\mathbb{P}}_{2}$, the Wasserstein distance between them is defined as follows:

(2) W(ℙ1,ℙ2)=infγ~∏(ℙ1,ℙ2)E(x,y)~[||x-y||]γ

where γ denotes the joint distribution of samples x and y, $\prod ({\mathbb{P}}_{1},{\mathbb{P}}_{2})$ denotes the set of all possible joint distributions of ${\mathbb{P}}_{1}$ and ${\mathbb{P}}_{2}$ combined, and ||x-y|| is the inter-sample distance. Based on the joint distribution γ, the expectation value of the distance between the sample pair is E(x,y)~[||x-y||]γ. Wasserstein distance between ${\mathbb{P}}_{1}$ and ${\mathbb{P}}_{2}$ is the infimum of the expectation value in all possible joint distributions. Using the Kantorovich-Rubinstein duality, Equation (2) can be transformed into:

(3) W(ℙ1,ℙ2)=sup||f||L≤1Ex~[f(x)]-Exℙ1[f(x)]~ℙ2

where ||f||L≤1 indicates that f is 1-Lipschitz continuous. 

### 2.5. Generalization of Emotional Representation in Adversarial Training

There are individual differences among speakers, such as timbre, expressive habits, etc., which make it difficult for the model to learn robust emotional representations that can cover all speakers. In order to generalize the emotional representations, we use adversarial training that forces the feature extractor to learn speaker-invariant emotional representations. We train the modules in the model alternatively, which consists of two steps: (1) training the discriminator; (2) training the feature extractor and emotion classifier. The details of these two steps are described in the following.

#### 2.5.1. Training of Discriminator

The discriminator is primarily responsible for estimating the Wasserstein distance between the distribution of the emotional representations of the source domain and the target domain speaker. We can represent all possible functions f in Equation (3) with discriminators since neural networks can fit various functions. Then the Wasserstein distance between the source domain and the target domain speakers can be calculated by:

(4) W(ℙs,ℙt)=sup||fD||L≤1Ex~[fd(fe(x))]-Ex~ℙs[fd(fe(x))]ℙt

where $f_{d}$ denotes the discriminator and $f_{e}$ denotes the feature extractor. To ensure that the discriminator function is 1-Lipschitz continuous, in [21], the authors propose to clip the weights of the discriminator within a compact space [−c, c] after each gradient update. However, Gulrajani et al. [32] found that weight clipping would result in gradient explosion or vanishing. In order to enhance the stability of gradients, Gulrajani et al. proposed the use of gradient penalties instead of weight clipping. As suggested in [32], gradient penalties are used to make the discriminator function 1-Lipschitz continuous in this paper. The gradient penalty term is defined as follows:

(5) GP=λEx~[||∇fd(fe(x))||2−1]2χ

where λ is the penalty factor, χ denotes the sample space distribution, and $\nabla f_{d}$ is the gradient of the discriminator.

Then the loss function ${ℒ}_{d}$ of the discriminator is shown as follows:

(6) ℒd=Ex~[fd(fe(xt))]ℙt−Ex~[fd(fe(xs))]ℙs+GP

The discriminator weight parameters are updated by minimizing Equation (6). In this training step, the weight parameters of the feature extractor and the emotion classifier are frozen.

#### 2.5.2. Training of Feature Extractor and Emotion Classifier

The feature extractor is responsible for extracting emotional representations under multiple data augmentations, and the emotion classifier gives the labels to which the representations belong. In this step, we force the feature extractor to learn the speaker-invariant emotional representations, and significant classifiable information is retained in those representations. The loss function ${ℒ}_{e}$ of the feature extractor and the loss function ${ℒ}_{c}$ of the emotion classifier are as follows:

(7) ℒe=Ex~[fd(fe(xs))]ℙs−Ex~[fd(fe(xt))]ℙt

(8) $${ℒ}_{c}=-\sum_{i}\sum_{c=1,x\in X^{s}}^{M}y_{ic}\log[f_{c}(f_{e}(x^{s}))]$$

In Equation (8), $f_{c}$ denotes the emotion classifier and $y_{ic}$ denotes the sample label. Then the joint loss function ${ℒ}_{ec}$ of the feature extractor and the emotion classifier is as follows:

(9) $${ℒ}_{ec}=\beta {ℒ}_{e}+{ℒ}_{c}$$

where β is the coefficient that controls the balance between discriminative and generalized representation learning. By minimizing Equation (9), the weight parameters of the feature extractor and emotion classifier are updated. In this step, the weight parameters of the discriminator are frozen.

Following adversarial training, a feature extractor that generalizes emotional representations while retaining classifiable information is developed, as well as an emotion classifier with superior classification performance.

## 3. Experiments

We evaluated the effectiveness of the proposed data augmentation methods in SER in Section 3.2. In Section 3.3, data augmentation methods that can significantly improve SER performance are applied to the proposed strong generalized speech emotion recognition model based on effective data augmentation.

### 3.1. Speech Emotion Corpus

To evaluate the proposed model, we conducted our experiments on Interactive Emotional Dyadic Motion Capture (IEMOCAP) [33]. The IEMOCAP contains 10,039 utterances annotated by at least three expert evaluators with a total length of approximately 12 h, which are divided into 9 emotions. There are five sessions in IEMOCAP, each with two speakers interacting (one male and one female).

To be consistent with previous studies, our experiment considered four emotions: happy, angry, sad, and neutral, and merged excitement into the happy class. The total number of utterances was 5531. Due to the variable length of each utterance, we segmented each utterance into two-second segments for extraction of log–Mel spectrograms. Log–Mel spectrogram features of each speech segment were extracted using 64 sets of Mel filters, 25 ms Hamming windows, and 10 ms window shifts. The experiments all adopted a speaker-independent strategy, i.e., a 5-fold Leave-One-Session-Out cross-validation strategy. For each fold, four sessions were selected for training and one session for testing. Weighted accuracy (WA) is used as a performance evaluation metric, which is commonly used in the SER field.

### 3.2. Experiment of Data Augmentation

In this section, a simple model is used to test the effects of the six data augmentation methods on SER under a variety of parameter settings.

The model used in this section consists of a feature extraction component and a classification component. The feature extraction component is the same as the sub-network setup in Section 2.3, and the classification component consists of fully connected layers and a softmax layer. A 1:1 ratio of original and augmented data amounts are used in the training set, and the test set data are not augmented. Adam optimizer is chosen with an initial learning rate of 1 × 10^−4^ and a weight decay of 1 × 10^−6^. The batch size is 64 and the number of training epochs is 50 in each fold.

#### 3.2.1. Speed Augmentation

We change the speed of the original speech to produce new speech to increase the training set. Table 1 shows the experimental results for the four speed factors. The first line of Table 1 shows that the WA of the model without data augmentation is 60.43%, and we use this result as the baseline. We first verified the impact of slow speech augmented in the training set on performance. Slowing down the speech rate has a small or no effect on performance improvement, according to the results. In subsequent experiments, two acceleration strategies are used. Performance is improved more significantly when speech speed is accelerated in addition to the training set. Compared to the baseline, WA improved by 2.01% and 0.93% at 1.5× and 2.0× speed, respectively.

#### 3.2.2. Noise Injection

To create new speech, we added White Gaussian Noise (WGN) with a mean of 0 and standard deviation of 1 to the original speech. We generated augmented speech by controlling the ratio of speech signal to WGN. Four signal-to-noise ratio (SNR) strategies were employed, and the experimental results are shown in Table 2. It should be noted that SNR here refers to the ratio of speech signal to WGN added. When the SNR was set to 30 dB, the WA decreased by 0.17% and improved by 0.99% when it was set to 60 dB. When the SNR was set to 90 dB and 120 dB, the WA also improved compared with the baseline. Noise interference will fade the spectrogram pattern, as shown in the spectrograms. As a result of the noise injection, the sentiment details are obscured to some extent, and it may appear that the performance improvement is not significant after augmentation at different SNR.

#### 3.2.3. Time Shifting

Time shifting refers to rolling the speech signal in the time domain. A total of four different time shifting strategies were compared, and the experimental results are shown in Table 3. With strategies of 60% and 80% shifting ratios, respectively, WA improved by 0.96% and 1.68% over the baseline. However, WA decreases at shifting ratios of 20% and 40%. As a result of rolling the speech signal, the overall coherence of speech is disrupted, which causes confusion for the classifier.

#### 3.2.4. Resampling

Resampling means changing the sampling rate of speech and creating re-sampled speech to augment the training set. We change the sampling rate of speech from 16,000 Hz to an intermediate sampling rate, and then back to 16,000 Hz. The experimental results are shown in Table 4 for four intermediate sampling rates. When the intermediate sampling rate is 11,000 Hz and 13,000 Hz, that is, when the intermediate sampling rate is smaller than the original sampling rate, WA performs better by 0.5% and 0.8%, respectively. As can be seen from the spectrogram, high-frequency details of speech are lost in these cases. A larger intermediate sampling rate was used in subsequent experiments. When the intermediate sampling rate was 18,000 Hz, WA improved by 1.4%. However, when the intermediate sampling rate was 20,000 Hz, WA decreased by 0.25%. As a result of changing the sampling rate of speech, some information is lost, which is a disadvantage for SER.

#### 3.2.5. Pitch Shifting

We use Python’s Librosa toolkit to change the pitch of the original speech. Pitch is altered by setting the parameters n_steps and bins_per_octave, where n_steps is how many steps to shift and bins_per_octave is how many steps per octave. A total of four different parameters were set, and the experimental results are presented in Table 5. Bins_per_octave was fixed at 12 in the first three experiments. As n_steps is set to 4 and 8, the WA is boosted by 2.42% and 0.56% respectively. When the pitch was adjusted downward, i.e., when n_steps was set to 6, the WA reached 63.60%, which is 3.17% higher than the baseline. A WA increase of 2.78% was observed when n_steps was set to 3 and the bins_per_octave was adjusted to 24 for the last experiment. Pitch shifting only changes the pitch without affecting the speed of speech, which can improve the generalizability of the data and the model to a certain extent. After changing the pitch, the waveform frequency increases, and the amplitude decreases. The corresponding spectrogram is more separable in frequency, i.e., the harmonics of speech can be separated more clearly, thus improving classification accuracy.

#### 3.2.6. Reverberation Augmentation

Reverberation is an effect that simulates the impulse response of a room to speech. We used the Pyroomacoustics toolkit in Python to add a reverberation effect to the original speech. It was decided that the spatial dimensions would be fixed at (10, 8, 3.5), which is similar to the dimensions of a real room. The reverberation time, the sound source location, and the microphone location were changed to generate six different reverberation effects. Table 6 presents the experimental results. During the first three experiments, we fixed the sound source and microphone position and only changed the reverberation time. Results indicated that higher or lower reverberation times could improve performance. The WA reached 63.05% when the reverberation time was set to 0.5 s, which is a 2.62% improvement over the baseline. Clearly, setting the reverberation time to 0.5 s is more appropriate. Following this, we alter the position of the sound source and microphone in space and fix the reverberation time. A WA of 61.26% was obtained when the sound source was placed at the edge of space and the microphone position was fixed. We then placed the microphone at the edge of space with the source at the previous position (3, 5, 1.75), and the WA reached 63.85%, an improvement of 3.42% compared to the baseline. We have achieved not only the highest level in reverberation augmentation, but the highest level in six of our proposed data augmentation methods. In the last experiment, we positioned both the sound source and microphone at the edge of space at a much farther distance from each other. The performance improvement was only 1.06% over the baseline, which was not satisfactory. As can be seen from spectrograms, after adding the reverberation effect to speech, there is a certain spread of frequencies. The more reverberation time there is, the more obvious the spread becomes. Moreover, the reverberation effect blurs the texture boundary of the spectrogram, making the correlation between adjacent frames stronger. This indicates that the superposition between signals after adding the reverberation effect makes emotional information in speech more apparent to some extent.

### 3.3. Experiment of the Strong Generalized Speech Emotion Recognition Model Based on Effective Data Augmentation

In this section, we apply the two best-performing data augmentation strategies from the experiments in the previous section to the strong generalized speech emotion recognition model based on effective data augmentation proposed in this paper.

We used pitch shifting and reverberation augmentation, and the most successful setting from the previous experiments was used. During training, the ratio of augmented data obtained by each data augmentation strategy and original data is 1:1. The feature extractor in the model contains three sub-networks, and the Log–Mel spectrogram of original speech, pitch shifting speech, and reverberation speech is input into each of the three sub-networks. The weighting coefficients of the sub-networks are set to 0.6, 0.2, and 0.2, respectively, and β is 0.6. The Adam optimizer is chosen with an initial learning rate of 1 × 10^−4^ and a weight decay of 1 × 10^−6^, and the learning rate is dynamically reduced during the training process according to model performance. The batch size is 32, and the number of training epochs in each fold is 50.

#### 3.3.1. Ablation Experiments

In Table 7 we report the performance of the strong generalized speech emotion recognition model based on effective data augmentation proposed in this paper. The WA is 66.51% under the speaker-independent experimental strategy. In order to verify the effectiveness of each module of the proposed model, three ablation experimental strategies are designed. (1) Without augmentation: no data augmentation is performed, and only the Log–Mel spectrogram features of the original speech are input. (2) Without multi-channel: the feature extractor has only one sub-network. (3) Without discriminator: remove the discriminator and do not generalize emotional representations. Table 7 presents the results of the ablation experiments. In the absence of data augmentation, WA decreases by 1.43%. With only one sub-network in the feature extractor, WA is also lower than that of the proposed model. This illustrates that the multi-channel feature extractor presented in this paper can effectively take advantage of multiple data augmentations to improve SER performance. When no discriminator is applied, WA decreases by 4.54%. It is evident from this that robust emotional representations are essential for SER. The results of ablation experiments show that the proposed model can further improve performance by using data augmentation on the basis of aligning the distribution of emotional representations.

#### 3.3.2. Comparison with Mainstream SER Algorithms

Additionally, the proposed model was compared with mainstream SER algorithms. Table 8 shows the results of the comparison of the WA of the algorithms obtained using a speaker-independent experimental strategy on the IEMOCAP corpus. By comparison with traditional algorithms such as SVM, HMM, and ELM, the proposed model in this paper is superior by 9.76%, 7.05%, and 2.31%, respectively. Furthermore, we compare algorithms that use deep learning. The model proposed in this paper leads by 3.01% when compared to the RNN algorithm incorporating the attention mechanism. Finally, we compare an algorithm that utilizes adversarial training to learn speaker-invariant emotional representations, and the algorithm also employs speed augmentation. Our proposed model still outperforms this algorithm by 7.89%, which is a significant improvement. It further demonstrates the superiority of the proposed model in terms of effective data augmentation and generalization of emotional representations.

#### 3.3.3. T-SNE Visualization of Emotional Representations

**Figure 4:**
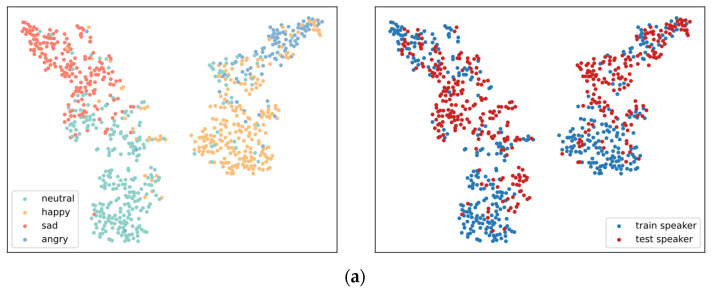

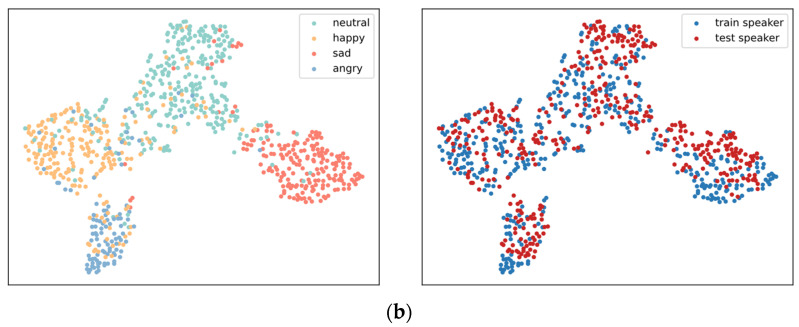
T-SNE visualization of emotional representations. (**a**) representations from the baseline model; (**b**) emotional representations from the proposed model.

We compared the learned emotional representations from the model proposed in this paper and the baseline model in Section 3.2. Both emotional representations were visualized as T-SNE [38] in Figure 4. The emotional representations from the baseline model have a low degree of inter-class separation; particularly, “happy” and “angry” are entangled in hidden space. Furthermore, there is a domain shift between the representations of the training set and test set speakers. In contrast, the emotional representations from the proposed model form four distinguishable clusters, i.e., there is a high degree of separation between classes. Even the representations of speakers from the test set show excellent inter-class discrimination. Additionally, the representation distributions are effectively aligned, allowing for the mixing of representations from different speakers. Accordingly, the proposed model is capable of generalizing emotional representations while maintaining the validity of the category information in the representations.

## 4. Conclusions

In this paper, we investigated the problem of data augmentation in SER and proposed a strong generalized speech emotion recognition model based on effective data augmentation. First, we evaluated the effectiveness of the six proposed data augmentation methods: speed augmentation, noise injection, time shifting, resampling, pitch shifting, and reverberation augmentation. The experimental results of data augmentation show that some attributes of speech can be detrimental to emotion recognition when they are changed. Injection of noise obscures the emotional details of speech to a certain extent, resampling causes the loss of information in speech, and time shifting disrupts the overall coherence of speech, all of which are detrimental to speech recognition. The experimental results indicate that pitch shifting and reverberation augmentation are the two most effective methods for improving SER performance. In the pitch shifting experiment, when bins_per_octave and n_steps were set to 12 and −6, respectively, WA was improved by 3.17% compared to the baseline results. When the pitch of speech is changed, the spectrogram is more separable in frequency, i.e., the harmonics of speech can be separated more clearly, thus improving the classification accuracy. In the reverberation augmentation experiment, WA was improved by 3.42% when the reverberation time, source location and microphone location were set to 0.5, (3, 5, 1.75) and (9, 7, 1.2), respectively. The superposition between the signals after adding the reverberation effect somehow makes the emotional information in speech more obvious.

Then, these two data augmentation strategies were applied to the model proposed in this paper. We conducted ablation experiments on the proposed model, and the results show that performance degradation is the greatest when the discriminator is not used. This indicates that individual differences among speakers are responsible for the performance degradation of SER and that the development of robust emotional representations is important for SER. The results of mainstream SER algorithms on the IEMOCAP corpus were compared with the proposed model. The WA of the proposed model is 2–9% higher than those of the relevant algorithms. According to the T-SNE visualization results, the representations from the proposed model exhibit better inter-class separability as well as generalization, which further proves the superiority of this work.

In future work, we may study the problem of representation generalization on cross-corpus SER. Performance tends to drop significantly in the case of cross-corpus, which is a challenging task. Moreover, we may consider modalities such as video and text to further improve the performance of emotion recognition.

## Figures and Tables

**Table 1:** Experimental results of speed augmentation. Speed factor represents the speed of augmented speech.

Speed Factor	WA (%)	Spectrogram
-	60.43	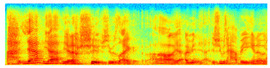
0.5	60.57	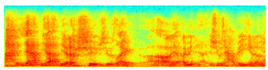
0.8	60.38	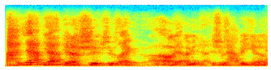
1.5	62.44	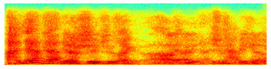
2.0	61.36	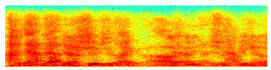

**Table 2:** Experimental results of noise injection. The SNR represents the ratio of speech signal to WGN added.

SNR	WA (%)	Spectrogram
-	60.43	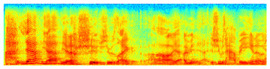
30 dB	60.26	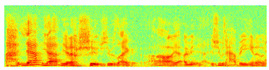
60 dB	61.42	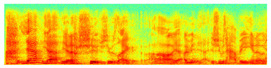
90 dB	60.44	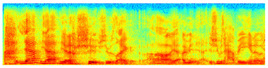
120 dB	61.25	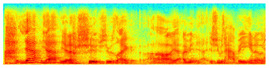

**Table 3:** Experimental results of time shifting. Shifting ratio represents the proportion of speech signal rolling in the time domain.

Shifting Ratio	WA (%)	Spectrogram
-	60.43	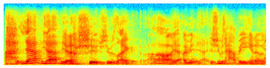
20%	59.73	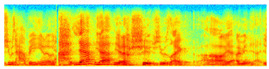
40%	60.29	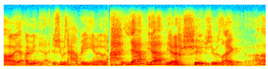
60%	61.39	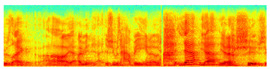
80%	62.11	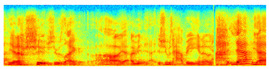

**Table 4:** Experimental results of resampling.

Intermediate Sampling Rate	WA (%)	Spectrogram
-	60.43	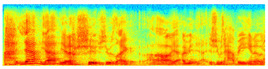
11,000 Hz	60.93	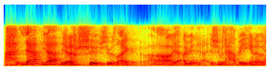
13,000 Hz	61.23	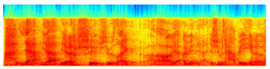
18,000 Hz	61.81	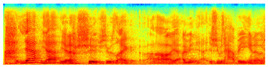
20,000 Hz	60.18	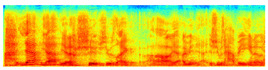

**Table 5:** Experimental results of pitch shifting.

Parameter 1 (Bins_per_octave)	Parameter 2 (n_steps)	WA (%)	Spectrogram
-	-	60.43	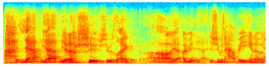
12	4	62.85	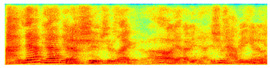
12	8	60.99	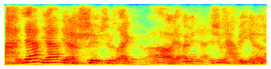
12	−6	63.60	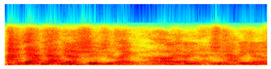
24	3	63.21	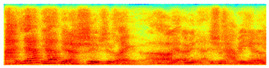

**Table 6:** Experimental results of reverberation augmentation. Time represents the reverberation time of speech, and Source and Microphone represent the positions of the sound source and microphone in the simulated space, respectively.

Time	Source	Microphone	WA (%)	Spectrogram
—	—	—	60.43	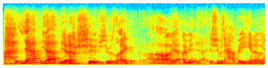
0.5	(3, 5, 1.75)	(7.5, 5.8, 1.2)	63.05	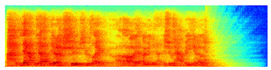
0.8	(3, 5, 1.75)	(7.5, 5.8, 1.2)	62.25	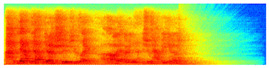
0.2	(3, 5, 1.75)	(7.5, 5.8, 1.2)	61.19	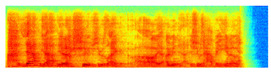
0.5	(1, 1, 1.75)	(7.5, 5.8, 1.2)	61.26	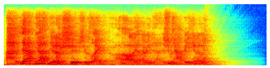
0.5	(3, 5, 1.75)	(9, 7, 1.2)	63.85	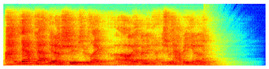
0.5	(1, 1, 1.75)	(9, 7, 1.2)	61.49	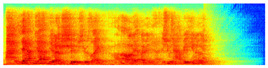

**Table 7:** Results of the ablation experiment.

Methods	WA (%)
Our proposed	66.51
Without augmentation	65.08
Without multi-channel	66.01
Without discriminator	61.97

**Table 8:** Performance of the proposed model and mainstream SER algorithms on the IEMOCAP corpus.

Methods	WA (%)
Our proposed	66.51
Lexical-Norm + SVM [34]	56.75
MEnAN + Speed augmentation [6]	58.62
MFCC + HMM [35]	59.46
RNN + Attention [36]	63.50
Region-switching + ELM [37]	64.20

## Data Availability

Publicly available datasets were analyzed in this study. This data can be found here: [IEMOCAP] [https://sail.usc.edu/iemocap/] (accessed on 29 December 2022).
